# TREM2-dependent activation of microglial cell protects photoreceptor cell during retinal degeneration via PPARγ and CD36

**DOI:** 10.1038/s41419-024-07002-z

**Published:** 2024-08-26

**Authors:** Wenchuan Zhou, Jincan He, Guiyan Shen, Ya Liu, Peiquan Zhao, Jing Li

**Affiliations:** 1 Department of Ophthalmology, Xinhua Hospital Affiliated to Shanghai Jiao Tong University School of Medicine, Shanghai, 200092 China; 2 Institute of Traditional Chinese Medicine and Stem Cell Research, College of Basic Medical Sciences, Chengdu University of Traditional Chinese Medicine, Chengdu, 610075 China

**Keywords:** Cell death and immune response, Chemotaxis, Microglia

## Abstract

Retinal degeneration is a collection of devastating conditions with progressive loss of vision which often lead to blindness. Research on retinal microglial cells offers great therapeutic potential in deterring the progression of degeneration. This study explored the mechanisms underlying the TREM2-mediated protective function of activated microglial cells during retinal degeneration. N-methyl-N-nitrosourea (MNU)-induced retinal degeneration was established in C57BL/6 J (WT) and *Trem2* knockout (*Trem2*^*−/−*^) mice. We discovered that MNU treatment led to the concurrent processes of photoreceptor apoptosis and microglia infiltration. A significant upregulation of disease-associated microglia signature genes was observed during photoreceptor degeneration. Following MNU treatment, *Trem2*^*−/−*^ mice showed exacerbated photoreceptor cell death, decreased microglia migration and phagocytosis, reduced microglial PPARγ activation and CD36 expression. Pharmaceutical activation of PPARγ promoted microglial migration, ameliorated photoreceptor degeneration and restored CD36 expression in MNU-treated *Trem2*^*−/−*^ mice. Inhibition of CD36 activity worsened photoreceptor degeneration in MNU-treated WT mice. Our findings suggested that the protective effect of microglia during retinal degeneration was dependent on *Trem2* expression and carried out via the activation of PPARγ and the consequent upregulation of CD36 expression. Our study linked TREM2 signaling with PPARγ activation, and provided a potential therapeutic target for the management of retinal degeneration.

## Introduction

Retinal degeneration is a collection of conditions characterized by the progressive loss of retinal neurons, mostly photoreceptor cells and to a lesser extent, retinal ganglion cells [1–3]. Inherited retinal degeneration frequently leads to blindness during early life stages in affected subjects, while the development of age-related retinal degeneration results from complex interactions among genetic, metabolic and aging-related factors [4, 5]. Regardless of the underlying causes of retinal degeneration, early detection and controlled clearance of dead or dying neurons, along with regulated inflammatory responses throughout the degeneration process, are crucial factors influencing disease progression. In this context, research on microglial cell responses during retinal degeneration holds significant importance.

The role of microglial cells during photoreceptor cell degeneration is complex and even controversial. Chemical or genetic depletion of microglial cells preserves photoreceptor cells in rd1, rd10, light- and retinal detachment-induced photoreceptor cell degeneration models [6–9]. However, the ablation of C3-C3R phagocytic mechanism in microglial cells accelerated photoreceptor cell death [10]. In rd1 mice during the post-rod cell degeneration phase, the phagocytic activity of microglial cells had little effect on the remaining cone cells [11, 12]. Additionally, microglial cells were not essential for prion disease-induced photoreceptor cell death [13, 14]. With the application of single-cell sequencing technique, distinct microglial cell populations were identified in brain and retina. One of the most-studied microglial cell subtype is disease-associated microglia (DAM) [15]. Initially identified in an animal model of Alzheimer’s disease (AD), DAM is characterized by reduced expression of homeostatic checkpoint genes such as *Tmem119*, *P2ry12*, and *Hexb*, and increased expression of genes associated with phagocytosis, lipid metabolism, and immune responses such as *Cstb*, *Lgals3*, *Lpl*, *Cst7*, *Cd9*, *Clec7a*, and *Cd68*. Further study suggests a two-step sequential activation of homeostatic microglial cell to DAM, with the final activation relying on Triggering Receptor Expressed on Myeloid cells 2 (TREM2), a cell surface receptor selectively expressed in microglial cells of the brain and retina. TREM2 stimulation promotes microglial cell chemotaxis, phagocytosis, survival and proliferation. The absence of TREM2 diminishes the characteristic features of DAM in degenerative brain conditions [16–18]. Depending on animal models and disease stages, both protective and detrimental effects of TREM2 were reported during neurodegeneration.

Despite extensive studies on microglial cell-expressed TREM2 in neurodegenerative conditions, its role during retinal degeneration remains inadequately explored. The existence of DAM-like microglial cells was suggested, with protective role on photoreceptor cells [19]. However, the mechanisms underlying the protective role remained unclear. In a previous study, we demonstrated that TREM2 protects photoreceptor cells in a mouse model of retinal detachment by limiting proinflammatory responses [20]. In this study, we utilized chemical-induced and inherited mouse models of retinal degeneration to investigate the characteristics of microglial cell activation and the role of TREM2-mediated microglial cell activity during the process. Our findings confirmed the significant upregulation of the DAM signatory genes in both models. Using *Trem2* knockout (*Trem2*^*−/−*^) mice, we showed that TREM2 is required for the expression of the DAM signatory genes, and TREM2-dependent microglial cell activation preserves photoreceptor cells during degeneration. Furthermore, we discovered that TREM2 upregulates CD36 expression by activating peroxisome proliferator-activated receptor gamma (PPARγ) signaling, thereby promoting microglial cell infiltration and mitigating photoreceptor degeneration. Taken together, our study underscored the importance of TREM2 in regulating microglia activation, and identified PPARγ signaling and CD36 as potential mechanisms and effector molecules of TREM2 in the context of retinal degeneration.

## Materials and methods

### Animal husbandry, the induction of retinal degeneration and treatments

The experiments conducted in this study adhered to the guidelines of the Association for Research in Vision and Ophthalmology Statement for the Use of Animals in Ophthalmic and Vision Research and were approved by the Institutional Animal Care and Use Committee of Xinhua Hospital Affiliated to Shanghai Jiao Tong University School of Medicine (Approval No. XHEC-F-2022-062). Wild-type C57BL/6J mice (WT) were purchased from Shanghai Jihui Animal Care Co., Ltd. (Shanghai, China), *Trem2*^*−/−*^ mice in C57BL/6J background were purchased from Shanghai Model Organisms Center Inc. (Shanghai, China), and rd10 (Pde6b^R560C^) mice were purchased from Gempharmatech Co. Ltd. (Nanjing, China). All animals were housed in a specific-pathogen-free mouse facility under a 12-hour light/12-hour dark cycle and *ad libitum* access to food. No randomization was performed.

Two mouse models of retinitis pigmentosa were included in this study. Mice homozygous for rd10 mutation at the age between postnatal 15 to 30 days (P15-30), which correspond to the onset and active progression of photoreceptor cell degeneration period, were used. Wild-type littermates were used as controls. MNU is a potent chemical mutagen that induces DNA damage specifically in the photoreceptors. MNU-induced photoreceptor cell degeneration was achieved through a single intraperitoneal injection of 1% MNU dissolved in PBS (60 mg/kg body weight) [21]. The controls were PBS-injected mice with the same genotype. Investigator was blinded while assessing the severity of retinal degeneration since genotype was carried out by another investigator. Except for rd10 mice, all other animals were used at the age between 8 and 10 weeks.

The following chemicals were procured from MedChemExpress, NJ, USA: GW1929 (HY-15655, a PPARγ agonist), GW9662 (HY-16578, a PPARγ antagonist), GW0742 (HY-13928, a PPARβ/δ agonist), sulfosuccinimidyl oleate sodium (SSO, HY-112847A, a CD36 inhibitor). GW1929 was dissolved in saline containing 10% dimethyl sulfoxide (DMSO) and 20% sulfobutylether-β-cyclodextrin (SBE-β-CD) at a concentration of 6 mg/mL, and administered to mice via intraperitoneal injection twice at the dose of 30 mg/kg body weight. GW9662 was dissolved in 10% DMSO and 90% corn oil at a concentration of 1 mg/mL and administered to mice via intraperitoneal injection twice at 4 mg/kg body weight. SSO was emulsified in 50% polyethylene glycol 300 at a concentration of 10 mg/mL and administered to mice via oral gavage at 50 mg/kg. GW0742 was dissolved in 10% DMSO and 90% corn oil at a concentration of 4 mg/mL and administered to mice via intraperitoneal injection twice at 20 mg/kg body weight.

### Retinal tissue processing, immunohistochemistry and terminal deoxynucleotidyl transferase dUTP nick end labeling (TUNEL) and lipid content staining

Mice were euthanized by sodium pentobarbital (120 mg/kg body weight) and their eyes were enucleated. After enucleation, the lens-free eyecup was fixed in 4% paraformaldehyde (PFA) for 1 hour at room temperature, and sequentially dehydrated in 20% and 40% sucrose at 4 °C. The processed tissue was embedded in optimal cutting temperature compound (OTC), and sectioned at a thickness of 10 μm using a microtome (CM1950, Leica Biosystems, Wetzlar, Germany).

Immunofluorescent staining of the frozen sections was carried out as previously described [20]. We used 10% goat or donkey serum for blocking, 0.2% Triton X-100 for permeabilization. The following primary antibodies: rabbit anti-IBA1 (Wako, #019-19741, 1:500), goat anti-IBA1 (Wako, #011-27991, 1:500), rat anti-CD68 (Bio-Rad, #MCA1957, 1:100), and rabbit anti-TREM2 (Abcam, #ab305103, 1:200) were used and incubated overnight at 4 °C. Alexa Fluor-488- or 568-conjugated secondary antibodies were purchased from ThermoFisher. Alexa Fluor^®^ 594 anti-mouse I-A/I-E Antibody (Biolegend, #107650, 1:200) was used for MHC class II (MHC II) protein staining. Finally, slides were sealed with an antifade reagent containing 4’,6-diamidino-2-phenylindole (DAPI) (ThermoFisher, #S36938).

TUNEL labeling (Roche, #11684795910), Oil Red O (ORO) and BODIPY staining (Beyotime, #C0158S and #C2053S) were performed according to the manufacturer’s instructions.

Fluorescent stained sections were imaged using either fluorescent microscopy (Olympus BX51) or confocal microscopy (Leica TCS SP8). Hematoxylin and eosin (H&E) stained sections were imaged using light microscopy. To determine the thickness of the outer nuclear layer (ONL), at least three sections from three different retinas were used, and five measurements were taken with equal spacing within each section. The average of all measurements within the same group and the standard deviation was presented.

### Enzyme-linked immunosorbent assay (ELISA) and western blot analysis

Retinas were lysed using a pre-cooled radioimmunoprecipitation assay (RIPA) buffer supplemented with protease and phosphatase inhibitors (Meilunbio, China), and protein concentration was determined. ELISA kits from Elabscience were used according to the manufacturer’s instructions to measure the concentrations of interleukin (IL)-6 (IL-6) (E-EL-M0044), IL-1β (E-EL-M0037), and IL-10 (E-EL-M0046).

For western blot analysis, 10 µg total lysates were separated by SDS-PAGE and transferred to polyvinylidene difluoride (PVDF) membrane (#1620177, Bio-Rad, Shanghai, China). QuickBlock (#P0252, Beyotime) was used for blocking. Primary and secondary antibodies used in Western blots were anti-CD36 (CST, #28109S, 1:1000), anti-TREM2 (Abcam, #ab305103, 1:1000), anti-AXL (Abcam, #ab215205, 1:1000), anti-CD11c (CST, #97585, 1:1000), anti-β-actin (Proteintech, #66009-1-Ig, 1:20000), HRP-conjugated goat anti-rabbit (Proteintech, #SA00001-2, 1:10000) and HRP-conjugated goat anti-mouse (Proteintech, #SA00001-1, 1:10000). Enhanced chemiluminescence (ECL) reagent was used for visualization under ChemiDoc imaging system (#1705061, Bio-Rad). The intensity of the bands was quantified and calculated using Image J. β-actin was used as loading control. The full length uncropped original western blots were presented in Supplemental Material 2.

### Retinal tissue dissociation, microglial cell purification and flow cytometry

After enucleation, the neuroretina tissue was carefully isolated and immersed in oxygenated Ames solution for 25 min at 37 °C in an enzyme mix (Neural tissue dissociation kit, Miltenyi Biotec, #130-094-802). The digestion process was stopped by adding Ames solution containing 5% BSA. Subsequently, the mixture was gently pipetted up and down to achieve a single-cell suspension.

For the enrichment of microglial cells, magnetic CD11b MicroBeads (Miltenyi Biotec, #130-093-634) and the matching column (Miltenyi Biotec, #130-042-401) were used. To optimize the purity of microglial cells, two columns were sequentially utilized for each preparation.

For flow cytometry, the above single-cell suspension was filtered through a 70-mm strainer, washed with PBS containing 1% FBS, stained with Annexin V-FITC and propidium iodine (PI) at room temperature according to the manufacturer’s instructions (Absin, #abs50001), and acquired on a BD FACSCelesta flow cytometer (Becton Dickinson, Milan, Italy) with BD FACSDiva software (version 8.0.1.1). Data were analyzed using FlowJo software (version 10.8.1).

### RNA sequencing and data analysis

The transcriptional profile of the whole retinal tissue was generated by bulk sequencing (Novogene Corporation, Inc). The total amount and the integrity of RNA were assessed using Bioanalyzer 2100 (Agilent Technologies, CA, USA). Libraries were prepared and qualified from each sample. Different libraries were pooled based on their effective concentrations and the desired amount of data for sequencing using the Illumina NovaSeq 6000 platform. Raw data is freely available in the Gene Expression Omnibus (GEO) database (https://www.ncbi.nlm.nih.gov/geo/) with the accession number of GSE270758. The sequencing data were normalized, and differentially expressed genes (DEGs) were identified using DESeq2 (Version 1.20.0) with the criteria of |log_2_Fold Change | > 1 and adjusted *p*-value < 0.05. We performed functional enrichment analyses using clusterProfiler (Version 3.8.1).

The transcriptional profiles of purified CD11b^+^ microglia were generated using low-input RNA sequencing (BGI Genomics Co. Ltd.). This method involves the amplification of cDNA using SMART combined with a transposase-based library construction technique. The DEGs were identified based on the same criteria as described above and analyzed accordingly.

Additionally, we retrieved sequencing data (GSE152474) from the GEO database. This database was generated using GPL24247 (Illumina NovaSeq 6000), and comprised 7 retinal samples from *Rho*^*P23H/WT*^ and 7 samples from littermate control mice. *Rho*^*P23H/WT*^ is an established mouse model of photoreceptor cell degeneration caused by the P23H mutation in Rho. This dataset contained mRNA profile of retina from *Rho*^*P23H/WT*^ and the wild-type littermates at P30. We applied a similar data analysis procedure as described above to analyze these data.

### RNA extraction, reverse transcription, and relative quantitative PCR (qPCR) analysis

For RNA extraction, TRIzol reagent (ThermoFisher Scientific, Waltham, MA, USA) in combination with RNA Clean & Concentrator™-5 (ZYMO Research, Irvine, CA, USA) were used. Reverse transcription and qPCR analysis were performed using PrimeScript™ RT and TB Green® Premix Ex Taq™ II (Takara, Japan). The gene expression levels relative to β-actin were calculated using the 2^−ΔΔCt^ method. The sequences of primers used in this study are listed in Table 1.

**Table 1:** Primer Sequences Used in this Study.

**Mouse-Trem2**-Forward: 5ʹ-CTGGAACCGTCACCATCACTC-3ʹ,
**Mouse-Trem2**-Reverse: 5ʹ-CGAAACTCGATGACTCCTCGG-3ʹ;
**Mouse-Cd36**-Forward: 5ʹ-ATGGGCTGTGATCGGAACTG-3ʹ,
**Mouse-Cd36**-Reverse: 5ʹ-GTCTTCCCAATAAGCATGTCTCC-3ʹ;
**Mouse-β-actin**-Forward: 5ʹ-GGCTGTATTCCCCTCCATCG-3ʹ,
**Mouse-β-actin**-Reverse: 5ʹ-CCAGTTGGTAACAATGCCATGT-3ʹ.

### Statistical analysis

Data quantitation was based on at least three biological replica and was presented as mean ± standard deviation (SD). Unpaired Student’ *t*-test was used for comparison between groups. Graphs were made using GraphPad Prism 8.0.2 (GraphPad Software Inc., San Diego, CA, USA). Statistical significance was accepted at *p* < 0.05.

## Results

### The progression of photoreceptor death and microglial cell activation in MNU-induced retinal degeneration

**Fig. 1:**
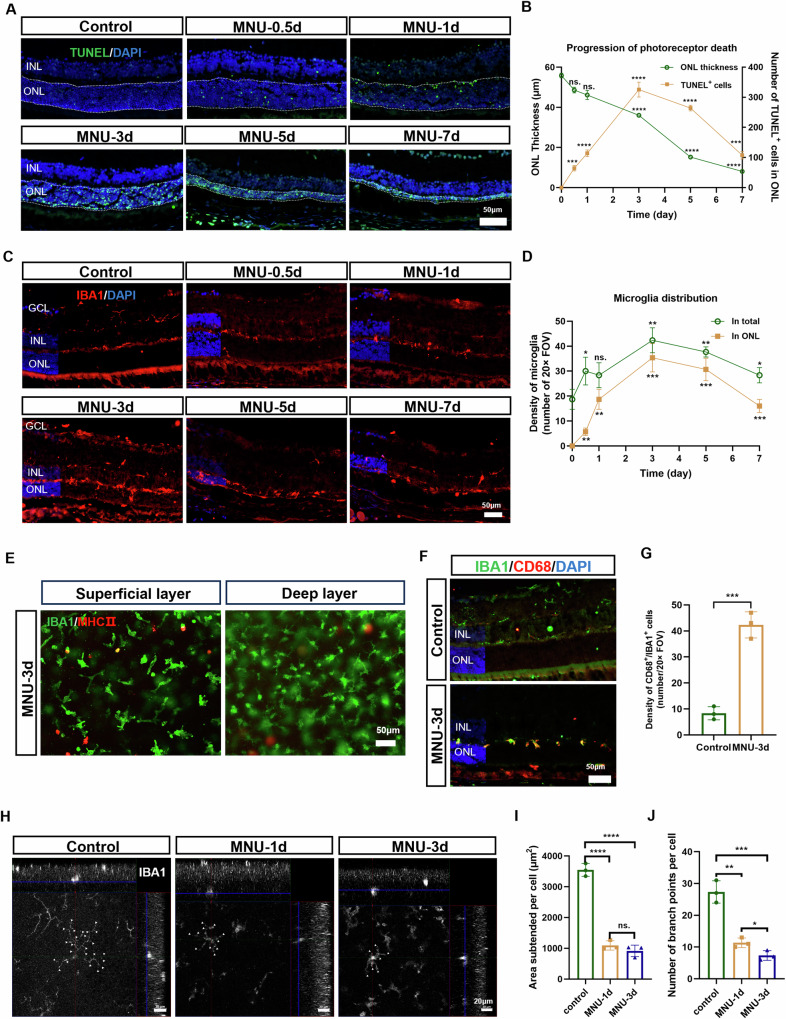
The progression of photoreceptor death and microglial cell activation in MNU-induced retinal degeneration. **A** Representative immunofluorescent images of TUNEL-stained retina during the progression of photoreceptor cell degeneration. Scale bar, 50 μm. **B** Quantitation of the number of TUNEL^+^ nuclei, represented as the density of TUNEL^+^ nuclei per 20× field of view, and the thickness of ONL during degeneration. **C** Representative immunofluorescent images of IBA1-stained cells during the progression of degeneration. Scale bar, 50 μm. **D** Quantitation of the number of infiltrated microglial cells, represented as the density of IBA1^+^ cell per 20× FOV in the ONL. **E** Representative immunofluorescent images of retinal wholemount (superficial layer and deep layer) showing the MHC II-positive peripheral phagocytes infiltrating into WT mouse retina. Scale bar, 50 μm. **F** Co-immunofluorescent staining of IBA1 and CD68. Scale bar, 50 μm. **G** Quantitation of IBA1- and CD68-positive infiltrating microglia. **H** Representative immunofluorescent images of microglia, showing the morphological changes. Scale bar, 20 μm. Quantitation of the subtended area (**I**) and branch points (**J**) of microglia within the ONL. *n* = 3 animals at each time point. INL, inner nuclear layer. ONL, outer nuclear layer. GCL, ganglion cell layer. Data and error bars indicate mean ± SD. ns, not significant. *p < 0.05, **p < 0.01, ***p < 0.001, and ****p < 0.0001.

To evaluate MNU-induced retinal degeneration, we analyzed the number of TUNEL^+^ nuclei and the thickness of the ONL at various time points following MNU treatment. TUNEL^+^ nuclei within the ONL were initially detected at 12 h post-MNU treatment, and the number of apoptotic cells increased progressively until day 3 before showing a decline. Concurrently, a significant thinning of the ONL was observed from day 3 and persisted until day 7 (Fig. 1A, B).

The number of IBA^+^ cells in the ONL exhibited a similar pattern to that of TUNEL^+^ photoreceptor cells. A few IBA^+^ cells were found in the ONL at 12 h post-MNU treatment. IBA^+^ cells peaked at day 3, and started to decline at day 5 (Fig. 1C, D). To discern the infiltration of peripheral phagocytes during the process, we stained the 3 days post-MNU treatment retina with MHC II. A few MHC II-positive cells were found in both superficial and deep layers of the degenerating retina, suggesting limited infiltration of peripheral phagocytes in MNU-induced retinal degeneration model (Fig. 1E). The results implicated that the IBA^+^ cells observed here were mainly microglia. The activated IBA1^+^ microglia also expressed CD68, indicating upregulated phagocytic activity (Fig. 1F, G). We also observed amoeboid change of these cells during the process with decreased branch points and subtended area (Fig. 1H–J). These findings suggested the concurrent activation and chemotaxis of resident microglia alongside photoreceptor death following MNU treatment.

### The expression of DAM signature during retinal degeneration

It is unclear whether photoreceptor cell degeneration activates microglial cells to DAM [19]. We addressed this issue by first examining the gene expression profiles of retinas collected from WT mice 3 days after MNU-treatment, as well as rd10 mice at postnatal days 15, 18, and 21.

**Fig. 2:**
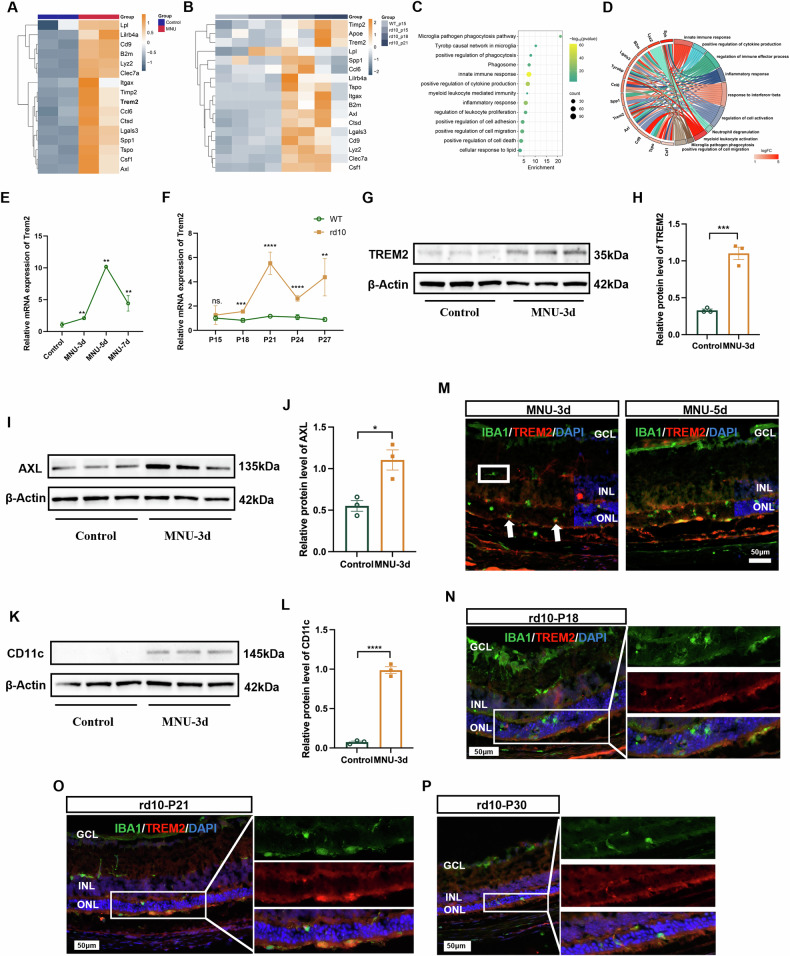
The expression of DAM signature during retinal degeneration. Heatmap illustrating DAM gene expression in MNU (**A**) and rd10 (**B**) mice. **C** Functional enrichment analysis of the upregulated genes in MNU-treated mice. **D** Chord diagram representing selected DEGs and the associated cellular processes, highlighting *Trem2* as a major modulator involved in most affected pathways during photoreceptor degeneration. qPCR results indicating increased *Trem2* expression in MNU-treated retina (**E**) and rd10 (**F**) retina. **G** Western blot for TREM2. **H** Quantitative analysis of the protein expression levels of TREM2 between control and MNU-3d mouse. **I** Western blot for AXL. **J** Quantitative analysis of the protein expression levels of AXL between control and MNU-3d mouse. **K** Western blot for CD11c. **L** Quantitative analysis of the protein expression levels of CD11c between control and MNU-3d mouse. **M** Co-immunofluorescent staining of TREM2 and IBA1 in MNU-treated retina revealing a faint signal of TREM2 in microglial cells outside the ONL (white square), contrasted with strong staining in cells at the ONL (white arrow). Scale bar, 50 μm. **N**–**P** Co-immunofluorescent staining of TREM2 and IBA1 in rd10 mouse retina demonstrating a strong signal of TREM2 in IBA1^+^ microglia infiltrating the ONL. Scale bar, 50 μm. Data were curated and presented as described in Fig. 1. Three to five animals were used for each time point in each group.

Increased expression of DAM signature genes including *Trem2*, *Axl*, *Lgals3*, *Itgax*, *Lpl* and *Cd9* was observed in MNU-treated retina (Fig. 2A). Similarly, increased expression of *Trem2*, *Apoe*, *Ctsd*, *Lgals3*, *Itgax*, and *Cd9* was found in P18 and P21 rd10 retina (Fig. 2B). To further explore the features of microglial cell activation during retinal degeneration, we analyzed the retinal gene expression profile of *Rho*^*P23H/WT*^ mouse (GSE152474 from GEO database). The results also showed the upregulation of characteristic DAM genes (Supplemental Material 1: Fig. S1). Functional enrichment analysis of the upregulated DEGs in MNU-treated mice revealed that the top GO terms were related to positive regulation of phagocytosis and inflammatory responses (Fig. 2C). Chord diagram showed that genes in TREM2 signaling (*Trem2*, *Tyrobp*, *Syk*) are key contributors to pathways affected (Fig. 2D) [22, 23].

The upregulation of *Trem2* was confirmed by qPCR analysis of MNU-treated WT and rd10 retinal samples (Fig. 2E, F). In addition, a significant upregulation of TREM2, AXL and CD11c (DAM signature) protein expression was also confirmed by western blot analysis of MNU-treated retina, compared to untreated controls (Fig. 2G–L).

To further investigate the distribution of activated microglial cells in the degenerative retina, retinal sections of MNU-treated mice were stained with TREM2 and IBA1. Strong TREM2 signal was exclusively found in microglial cells located in the ONL and subretinal space (Fig. 2M). Similarly, intense immunofluorescent staining of TREM2 was detected in microglial cells in the ONL and subretinal space of P18-P30 rd10 mice, but not in the inner retina (Fig. 2N–P). These findings collectively suggested that during the degeneration of photoreceptor cells, a subset of DAM-like microglia tended to aggregate at the ONL and subretinal space.

### *Trem2* deficiency deterred microglial infiltration, exacerbated photoreceptor cell death, and aggravated retinal inflammation during retinal degeneration

**Fig. 3:**
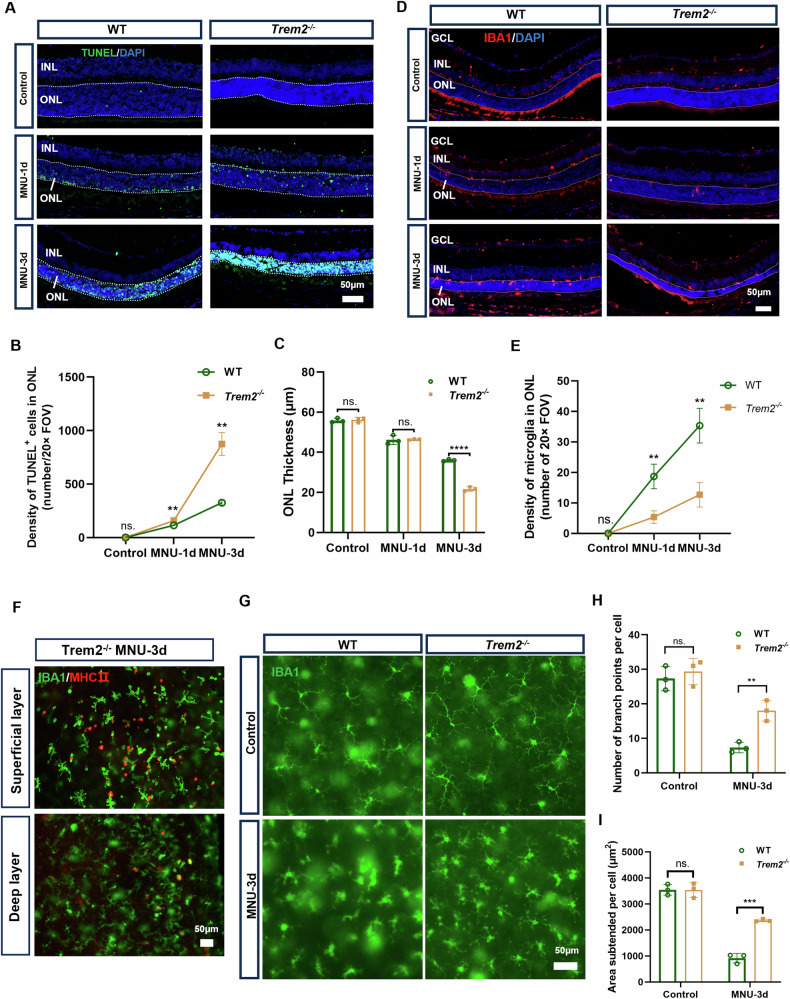
*Trem2* deficiency exacerbated photoreceptor cell death and deterred microglial infiltration in MNU-induced photoreceptor degeneration mice. **A** Representative immunofluorescent images of TUNEL-stained retina in WT and *Trem2*^*−/−*^ mice 1 and 3 days post-MNU treatment. Scale bar, 50 μm. Quantitative analysis of the number of TUNEL^+^ nuclei (**B**) and ONL thickness (**C**) in WT and *Trem2*^*−/−*^ mouse retina at 1 and 3 days post-MNU treatment. **D** Representative immunofluorescent images of IBA1-stained cells in WT and *Trem2*^*−/−*^ mice at 1 and 3 days post-MNU treatment. Scale bar, 50 μm. **E** Quantitative analysis of the density of IBA1^+^ cells in ONL in WT and *Trem2*^*−/−*^ mouse retina at 1 and 3 days post-MNU treatment. **F** Representative immunofluorescent images of retinal wholemount (superficial layer and deep layer) showing the MHC II-positive peripheral phagocytes infiltrating into *Trem2*^*−/−*^ mouse retina. Scale bar, 50 μm. **G** Representative immunofluorescent images of microglia showing the morphological changes at 3 days post-MNU treatment in WT and *Trem2*^*−/−*^ mice. Scale bar, 50 μm. Quantitative analysis of the number of branch points (**H**) and subtended area (**I**) of microglia in WT and *Trem2*^*−/−*^ mouse retina at 1 and 3 days post-MNU treatment. Data was curated and presented as described in Fig. 1. Three to five animals were used for each time point in each group.

To further elucidate the role of TREM2 in microglial cell activation and function during retinal degeneration, we induced photoreceptor degeneration using MNU in *Trem2*^*−/−*^ mice. Quantitative analysis of the ONL thickness and TUNEL^+^ photoreceptors revealed significantly more TUNEL^+^ photoreceptor cells and thinner ONL in the MNU-treated *Trem2*^*−/−*^ compared to MNU-treated WT retina, suggesting that *Trem2* deficiency exacerbated photoreceptor degeneration (Fig. 3A–C). At the meantime, fewer IBA1^+^ cells were found in the ONL of MNU-treated *Trem2*^*−/−*^ mice (Fig. 3D, E). To discern the participation of infiltrating macrophages, we performed IBA and MHC II co-staining and found slightly more MHC II^+^ cells in MNU-treated *Trem2*^*−/−*^ mice than in the MNU-treated WT mice at day 3, and these cells predominantly located at the superficial layer (Figs. 3F and 1E). However, it was clear that microglial cells dominated the IBA1^+^ cells, particularly in the ONL. Therefore, the results suggested that *Trem2* knockout attenuated the migration of microglial cells towards degenerating neurons. Additionally, the *Trem2*-deficient microglia displayed less amoeboid phenotype with more branch points and larger subtended area (Fig. 3G–I). Collectively, the results suggest that *Trem2* deficiency attenuated the migratory activity of microglial cells and exacerbated photoreceptor cell degeneration.

**Fig. 4:**
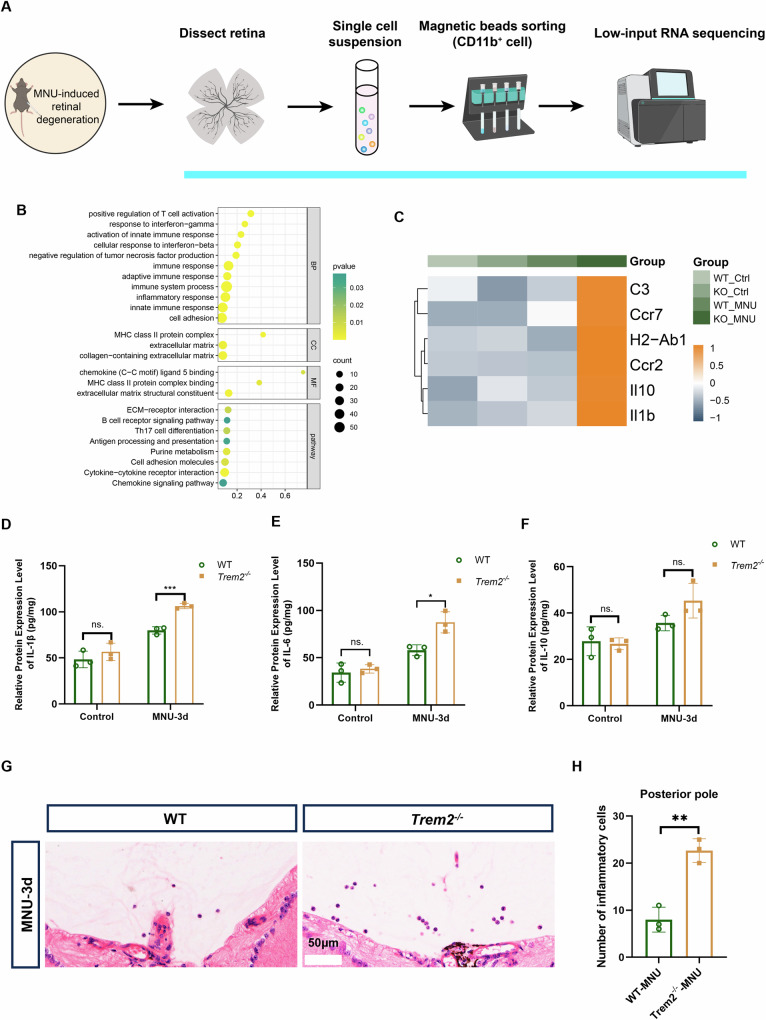
*Trem2* ablation aggravated the inflammatory responses in MNU-induced photoreceptor degeneration. **A** Flow chart illustrating microglial purification and low-input RNA sequencing. **B** Functional enrichment analysis of upregulated genes in microglia of MNU-treated *Trem2*^*−/−*^ retina. **C** Heatmap depicting the representative upregulated proinflammatory genes in microglia of the *Trem2*^*−/−*^ mice 3 days after MNU treatment. Relative protein levels (pg/mg of total protein) of IL-1β (**D**), IL-6 (**E**) and IL-10 (**F**) in total retinal lysates measured by ELISA. **G** Representative H&E staining of retinal sections from MNU-treated WT and *Trem2*^*−/−*^ mice. **H** Number of inflammatory cells in the vitreous cavity in MNU-treated WT and *Trem2*^*−/−*^ retina. Data was curated and presented as described in Fig. 1. Three animals were used for each time point in each group.

The inflammatory responses of WT and *Trem2*-deficient microglial cells were compared using cells enriched by magnetic CD11b microbeads from retina of the MNU-treated WT and *Trem2*^*−/−*^ mice (Fig. 4A). Because of the limited number of infiltrated cells, we believe the data largely reflected the expression profile of microglial cells. The upregulated genes in *Trem2*-deficient microglia were notably enriched in the chemokine signaling pathway, MHC II protein complex, and inflammatory responses (Fig. 4B). We observed higher mRNA levels of inflammation-related genes, such as *H2-Ab1*, *Il10*, and *Il1b*, in *Trem2*-deficient microglia (Fig. 4C). The changes in gene expression were selectively validated by ELISA analysis on total retinal lysates, including IL-1β, IL-6, and IL-10 (Fig. 4D–F). Consistently, we also found more inflammatory cells in the vitreous cavity of MNU-treated *Trem2*^*−/−*^ mice compared to MNU-treated WT mice by H&E staining (Fig. 4G, H). Taken together, these findings suggested that *Trem2* deficiency aggravated retinal inflammatory responses.

### *Trem2*-deficient microglia in degenerative retina showed distinct gene expression alteration

To unveil the underlying mechanism associated with *Trem2*-deficient microglia and aggravated photoreceptor death during retinal degeneration, we analyzed the downregulated genes in CD11b microbeads-enriched microglial cells of MNU-treated *Trem2*^*−/−*^ retina (relative to MNU-treated WT retina).

**Fig. 5:**
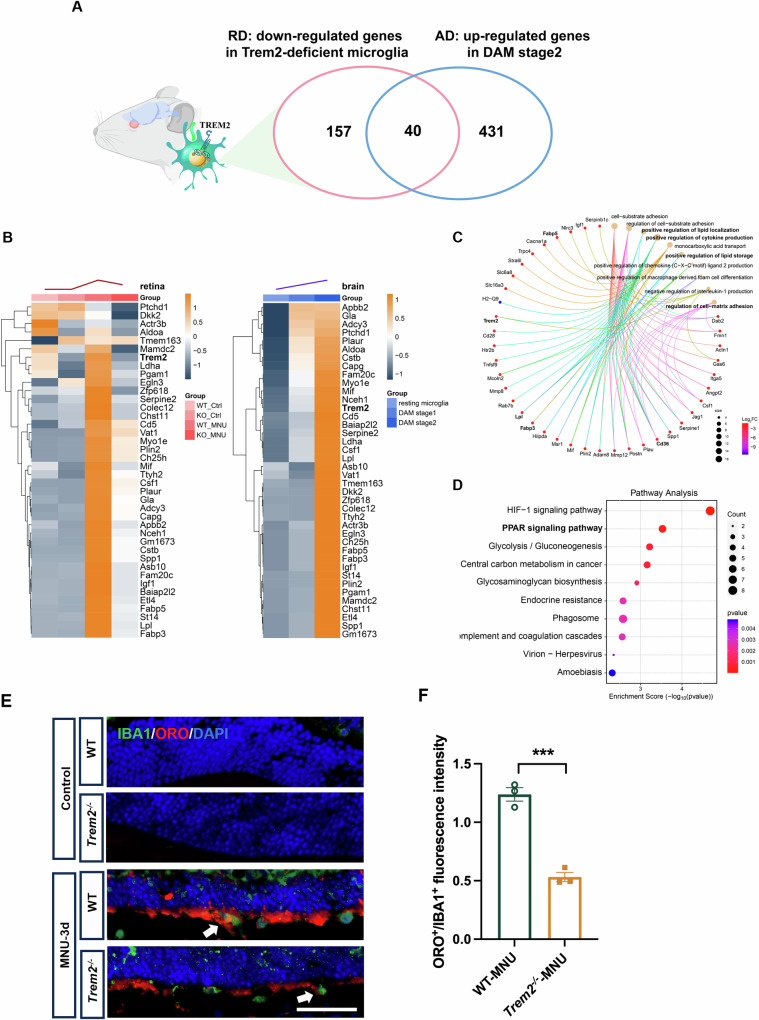
*Trem2*-deficient microglia in degenerative retina showed distinct gene expression alterations. **A** Venn graph showing the common genes between DAM markers from the AD datasets and downregulated DEGs in *Trem2*-deficient microglia 3 days post-MNU treatment. **B** Heatmap depicting the expression of 40 common genes between DAM markers from the AD datasets and downregulated DEGs in Trem2-deficient microglia 3 days post-MNU treatment. GO enrichment analysis (**C**) and Pathway analysis (**D**) of the downregulated genes in MNU-treated *Trem2*^*−/−*^ microglia compared to MNU-treated WT microglia. **E** Oil red O (ORO) staining of retinal sections from MNU-induced WT and *Trem2*^*−/−*^ mouse. **F** Quantitative analysis of ORO^+^/IBA1^+^ fluorescence intensity between WT and *Trem2*^*−/−*^ mouse. Data was curated and presented as described in Fig. 1. Three animals were used for each time point in each group.

When the downregulated genes in microglia of MNU-treated *Trem2*^*−/−*^ retina was compared with markers of DAM identified in the brain of AD mouse [15], a group of 40 genes were found in common, including *Spp1*, *Lpl*, *Mif*, *Fabp3* and *Fabp5*, which are related to lipid metabolism and phagocytosis (Fig. 5A, B). The result further supported the existence of DAM-like microglia during photoreceptor degeneration (Fig. 2). Further analysis of the downregulated genes showed the enrichment in lipid binding, transportation and storage, cell-substrate adhesion, cell-matrix adhesion, lysosome and phagocytic vesicle (Fig. 5C). KEGG analysis suggested the dysregulated HIF-1 signaling pathway, PPAR signaling pathway and phagosome among others (Fig. 5D). TREM2-dependent HIF-1 signaling was reported in microglial cells of 5XFAD mouse [24, 25]. PPAR signaling pathway is an important regulatory mechanism of lipid metabolism [26]. Genes including *Lpl*, *Fabp5*, *Fabp3* and *Cd36* are associated with PPAR signaling. We compared intracellular lipid content of microglial cells in the untreated and MNU-treated WT and *Trem2*^*−/−*^ retina using oil red O (Fig. 5E, F) and BODIPY staining (Supplemental Material 1: Fig. S2). Lipid accumulation was observed in MNU-treated microglial cells by both staining methods. However, it was significantly less in the MNU-treated Trem2^−/−^ microglial cells than the MNU-treated WT cells. These results further supported TREM2-related dysregulation of lipid metabolism.

### Pharmacological targeting of PPARγ promoted microglia infiltration and ameliorated photoreceptor degeneration in MNU-treated *Trem2*^*−/−*^ mice

To further explore the role of PPAR signaling in mediating TREM2-related microglial cell activation, we treated the MNU-treated WT and *Trem2*^*−/−*^ mice with GW0742, a PPARβ/δ agonist, and GW1929, a PPARγ agonist, and analyzed the changes in photoreceptor cell death and microglial cell activation.

**Fig. 6:**
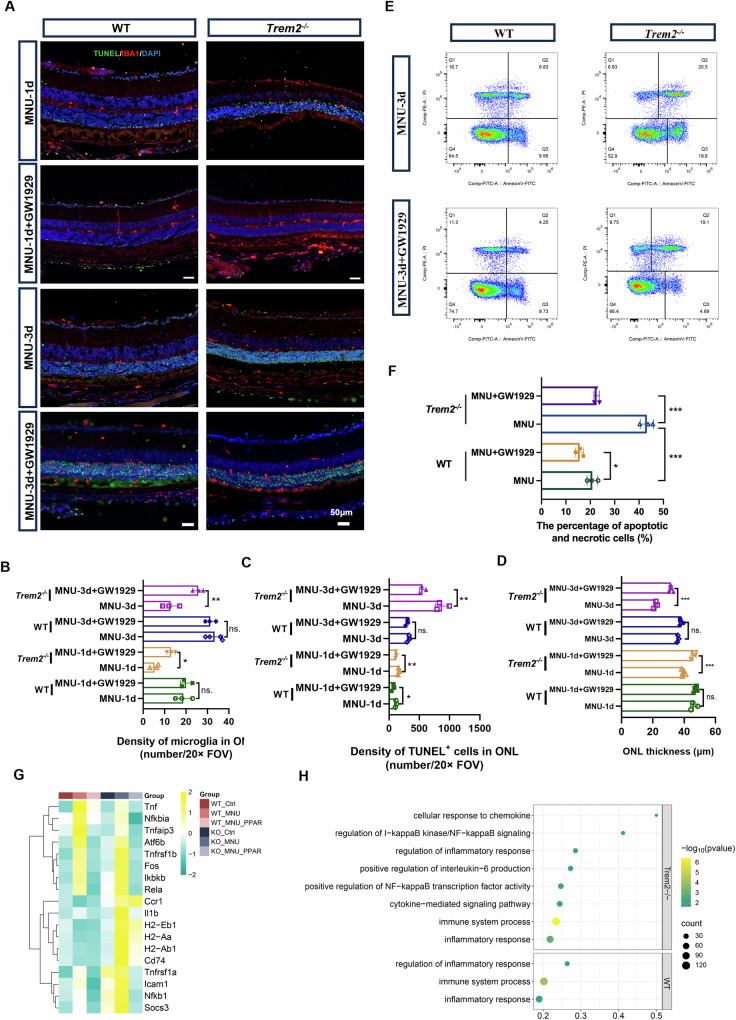
Pharmacological targeting of PPARγ promoted microglia infiltration and ameliorated photoreceptor degeneration in MNU-induced retinal degeneration. **A** Representative immunofluorescent images of TUNEL and IBA1-stained retina at 1 and 3 days post-MNU treatment in WT and *Trem2*^*−/−*^ mouse with intraperitoneal injection of PPARγ agonist (GW1929). Scale bar, 50 μm. The density of IBA1^+^ cells in ONL (**B**), the number of TUNEL^+^ nuclei (**C**), and ONL thickness (**D**) in WT and *Trem2*^*−/−*^ mouse retina at 1 and 3 days post-MNU treatment and PPARγ agonist treatment. **E** Flow cytometry analysis using Annexin V/PI staining. **F** Quantitative analysis of the number of apoptotic and necrotic cells (Annexin V^+^) between WT and *Trem2*^*−/−*^ mouse. **G** Heatmap displaying proinflammatory gene expressions with and without PPARγ agonist treatment in MNU-treated WT and *Trem2*^*−/−*^ retina. **H** Pathway analysis of downregulated genes after PPARγ agonist treatment in MNU-treated *Trem2*^*−/−*^ retina.

No significant changes in the number of microglial cells migrating towards the ONL was found in MNU-treated WT or *Trem2*^*−/−*^ mice following GW0742 treatment, suggesting that PPARβ/δ signaling did not play a significant role in this process (Supplemental Material 1: Fig. S3). On the other hand, intraperitoneal injection of GW1929 in MNU-treated *Trem2*^*−/−*^ mice significantly promoted microglia cell migration to the ONL and subretinal space, reduced the number of TUNEL^+^ cells and preserved the thickness of ONL (Fig. 6A–D). The reduction of apoptotic cells was also confirmed by flow cytometry analysis using Annexin V and PI as markers. Three days after MNU treatment, the *Trem2*^*−/−*^ retina showed significantly more Annexin V^+^ cells compared to WT (Fig. 6E, F). GW1929 treatment significantly reduced the percentage of Annexin V^+^ cells in the MNU-treated *Trem2*^*−/−*^ mice, while it had a small effect on WT mice (Fig. 6E, F).

To further test the role of microglial cell PPARγ signaling on photoreceptor cell death, we treated the MNU-treated WT mice with GW9662, a PPARγ inhibitor. Administration of GW9662 caused exacerbated photoreceptor cell death and ONL thinning (Supplemental Material 1: Fig. S4), further supporting that PPARγ signaling in microglial cells mediated the protective effect of microglia during retinal degeneration.

We also purified microglial cells from day 3 MNU-treated *Trem2*^*−/−*^ mice with and without GW1929 treatment, and compared their gene expression profiles. GW1929 treatment decreased the expression of genes encoding proinflammatory factors and MHC II molecules (Fig. 6G, H), suggesting that stimulation of PPARγ signaling abated the proinflammatory responses of microglial cells. Collectively, these results suggested that PPARγ signaling confers the protective role of microglial cells during retinal degeneration.

### PPARγ activation regulated CD36 expression, facilitated microglial migration into degenerative neurons

**Fig. 7:**
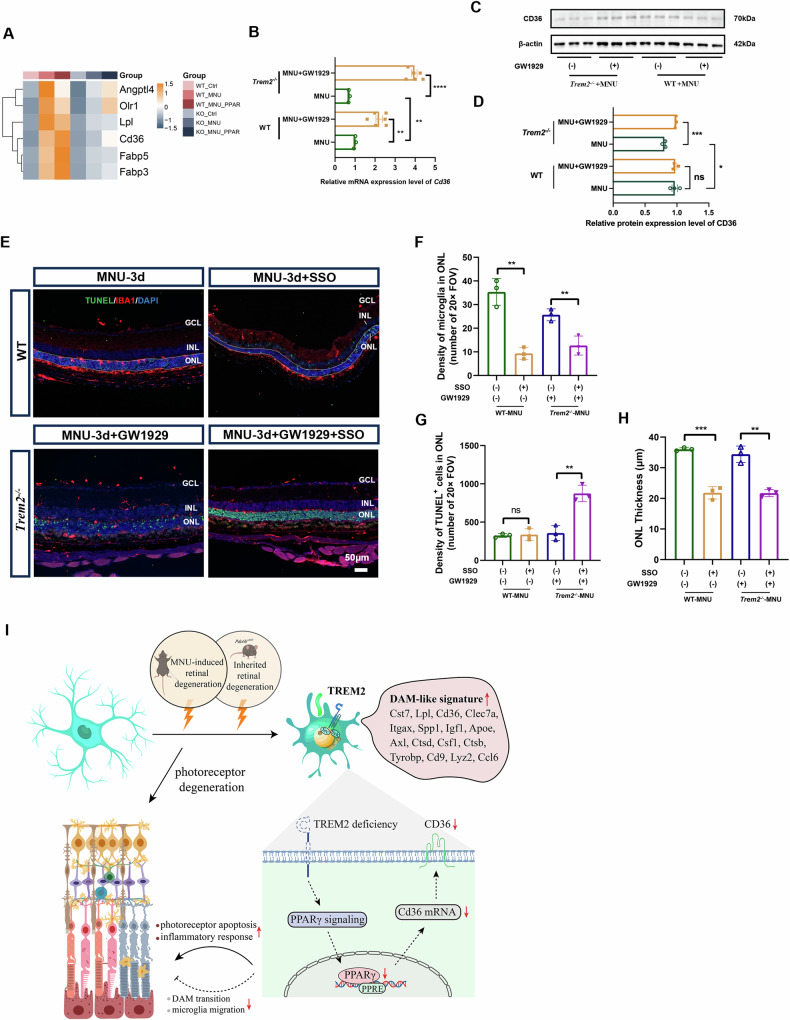
PPARγ activation stimulated *Cd36* expression and microglial cell migration into the degenerative photoreceptor cell layer. **A** Heatmap showing selective PPARγ target gene expression in *Trem2*^*−/−*^ microglia. **B** Quantitative analysis of *Cd36* gene expression with and without GW1929 treatment in MNU-treated *Trem2*^*−/−*^ and WT mouse retina. **C** Western blotting for CD36 in MNU-treated *Trem2*^*−/−*^ and WT mouse retina. **D** Quantitative analysis of CD36 protein expression levels in MNU-treated *Trem2*^*−/−*^ and WT mouse retina with and without GW1929 treatment. **E** Representative immunofluorescent images of TUNEL- and IBA1-stained retina of MNU-treated WT retina with and without CD36 inhibitor (SSO), and MNU-treated *Trem2*^*−/−*^ mice with PPARγ agonist (GW1929) and CD36 inhibitor (SSO). Scale bar, 50 μm. Quantitative analysis of microglial cell infiltration (**F**), the number of TUNEL^+^ nuclei (**G**) and the ONL thickness (**H**) in SSO-treated MNU-induced WT mouse retina and GW1929 + SSO treated MNU-induced *Trem2*^*−/−*^ mouse retina. **I** Schematic diagram depicts that the protective functions of TREM2-mediated microglia in retinal degeneration are carried out via the activation of PPARγ and the consequent upregulation of CD36 expression.

The target genes of PPARγ vary depending on the activating ligands, cell type and tissue context [27–29]. In microglial cells of MNU-treated *Trem2*^*−/−*^ retina, enhanced PPAR**γ** signaling upregulated lipid-related genes, such as *Cd36* and *Lpl* (Fig. 7A). We then examined the mRNA and protein expression of CD36 in MNU-treated WT and *Trem2*^*−/−*^ mice with and without PPAR**γ** agonist (GW1929) treatment (Fig. 7B–D). At both mRNA and protein levels, reduced CD36 expression was observed in MNU-treated *Trem2*^*−/−*^ mice compared to MNU-treated WT. GW1929 increased CD36 expression in MNU-treated *Trem2*^*−/−*^ mice. However, in MNU-treated WT mice, only a mild increase of CD36 mRNA was observed after GW1929 treatment. The relatively faint western blot band of CD36 likely reflected the small number of CD36-expressing cells in the retina.

Since CD36 is a scavenger receptor with both phagocytosis and lipid transport activities, we decided to test if it is the downstream effector of PPARγ signaling mediating the protective role of microglial cells during photoreceptor degeneration. MNU-treated WT mice was given SSO, an irreversible CD36 inhibitor. SSO treatment reduced microglial migration into the apoptotic photoreceptor cell layer and exacerbated ONL thinning (Fig. 7E). Co-administration of GW1929 and SSO to MNU-treated *Trem2*^*−/−*^ mice decreased microglial infiltration and accelerated photoreceptor degeneration compared to GW1929 treatment alone (Fig. 7F–H). The change of apoptotic cell number was also confirmed by flow cytometry analysis (Supplemental Material 1: Fig. S5). These results collectively suggested that PPARγ and CD36 are important effector molecules of TREM2, contributing to the protective function of activated microglial cells during photoreceptor cell degeneration (Fig. 7I).

## Discussion

Retinal degeneration encompasses a range of debilitating conditions that frequently result in significant vision impairment, even blindness. Irrespective of the underlying causes, microglial cells are central in regulating the progression of degeneration. In the present study, we showed the indispensable role of TREM2 in microglia activation and its protective effect during photoreceptor cell degeneration. Heightened microglial cell proinflammatory responses and photoreceptor cell death were found in mouse models of retinal degeneration in the absence of *Trem2*. We further demonstrated that the underlying mechanism was related to the activation of PPARγ signaling, and the subsequent upregulation of CD36 expression. Inhibition of PPARγ signaling or CD36 activity diminished the protective role of microglial cells on photoreceptor cells. Conversely, pharmacological activation of PPARγ compensated for the defective neuroprotective role of microglial cells in TREM2 deficiency. Additionally, our study also provided evidence to support the existence of DAM-like microglia during retinal degeneration. A schematic diagram is presented in Fig. 7I.

Although controversial reports existed, it is widely recognized that TREM2 in DAM exerts protective functions primarily through enhanced phagocytosis and lipid metabolism [30]. However, the downstream pathways through which TREM2 operates in DAM remain to be fully elucidated. Out study identified PPARγ as an important mediator of TREM2 activation in microglial cells during retinal degeneration. While this is likely the first report which demonstrates the direct involvement of PPARγ signaling in TREM2 function in retinal microglial cells, circumstantial evidence existed to support such a relationship. For example, studies on tissue-resident macrophages have shown that TREM2 and its downstream signaling pathways are part of a conserved mechanism for lipid sensing and response, suggesting that the role of TREM2 in modulating microglial activation is linked, at least in part, to its influence on lipid metabolism [17, 18]. Beyond the CNS, macrophages with increased TREM2 expression and concurrent upregulation of genes related to lipid metabolism and phagocytosis were found in the adipose tissue, liver and aorta [31, 32], suggesting potential crosstalk between TREM2 and PPARγ signaling. In fact, increased PPARγ and CD36 gene expressions were found in the white adipose tissue of *Trem2* transgenic mice [33]. Moreover, dysregulated PPARγ/p38 MAPK signaling and significant metabolic deficits, including impaired mitochondrial respiratory capacity and glycolytic immunometabolic switching, were observed in iPS-derived microglia from patients with loss-of-function *TREM2* variants [34]. Future investigations on the mechanisms connecting TREM2 signaling to PPARγ activation would not only validate our current findings but also enhance our understanding of TREM2’s role in regulating lipid metabolism in microglial cells.

Our study further suggested that CD36, a scavenger receptor originally known as fatty acid translocase, is an important effector molecule of PPARγ activation in retinal microglial cells. The activation of PPARγ led to increased *Cd36* expression, which rescued the dysfunctional microglia in *Trem2*^*−/−*^ mice by stimulating its migration towards degenerative photoreceptor cells and preventing the accelerated photoreceptor cell death. Although the systemic administration of GW1929 and SSO may affect other cells of the retina, for example, the macrophages and RPE cells. The effect contributed by these cells were likely not substantial. First of all, MHC II staining showed limited macrophage infiltration in the ONL and subretinal area of MNU-treated retina. Since RPE cells mainly engulf photoreceptor outer segment, the inhibition of its activity should have limited effect on ONL thickness, even though CD36 represents an important phagocytic mechanism in RPE [35, 36]. Therefore, we believe that the changes observed upon GW1929 and SSO treatment were mostly carried out by microglial cells.

The regulatory relationship between PPARγ and CD36 in microglial cells has been reported under various degenerative conditions [37, 38]. CD36-mediated myelin uptake by phagocytes (including macrophages and microglial cells) is necessary for CNS [39–41]. Microglial cells in amyloid precursor protein/presenilin 1 (APP/PS1) transgenic mice also exhibit PPARγ-induced CD36 upregulation, enhancing beta-amyloid uptake [42]. On the other hand, CD36 can also trigger proinflammatory effect through interaction with Toll-like receptors [43]. In retina, previous studies reported the role of CD36 in mitigating the proinflammatory responses of the mononuclear phagocytes (MNPs) in *Cx3cr1*^−/−^ and light-induced mouse models of retinal degeneration [44, 45]. Our study suggested that the dominant function of TREM2-mediated upregulation of CD36 is to enhance the migration and phagocytic activity of microglia. The absence of this mechanism in *Trem2*^*−/−*^ mice result in the accumulation of apoptotic photoreceptor cells, a likely consequence of insufficient clearance of dying cells at the onset of the degeneration. Of note, we observed a small increase of *Cd36* expression in MNU-treated *Trem2*^*−/−*^ mice compared to MNU-treated WT mice following GW1929 treatment. This observation suggested that, in the absence of CD36 activation, alternative compensatory mechanisms might be activated in *Trem2*-deficient microglia during photoreceptor degeneration. For example, recent research has shown that TREM2-mediated upregulation of galectin-3, also contributes to the phagocytic activity of microglial cells during retinal degeneration [46]. In fact, we observed significant downregulation of *Lgals3* in MNU-treated *Trem2*^*−/−*^ retina compared to MNU-treated WT retina, but not in mice treated with GW1929 during degeneration.

A limitation of this study is the lack of precisely distinguishing macrophages (monocytes-derived and ocular resident) from microglial cells. Although immunostaining of MHC II has been proven to label macrophages but not microglia in the retina, using monocyte- and microglia-specific tracing mice alongside microglia-specific *Trem2* knockout mice would provide more definitive answer [47, 48]. However, since only limited number of infiltrated macrophages were identified through MHC II staining, we believe that the observations in this study predominantly reflected the activity of microglial cells. A recent study identified disease inflammatory macrophages (DIMs), as a subset of microglial cells derived from monocytes with DAM-like features in aging and neurodegenerative brains [49]. Considering the contexts in which DIMs were identified, we speculate that they originate from the remaining monocytes following previous assaults (often seen in aging or degenerative neural tissue) [48]. Given that the mice we used here had no prior history of stress, it is unlikely that DIMs constitute a significant part of DAM in the present study. However, as discussed above, the contribution of non-microglial phagocytic cells during retinal degeneration needs to be further delineated.

### Supplementary information

Supplemental Material 1

Supplemental Material 2

## Data Availability

The datasets used and/or analyzed during the current study are available from the corresponding author on reasonable request.
