# Supplementary material for: TREM2-dependent activation of microglial cell protects photoreceptor cell during retinal degeneration via PPARγ and CD36
Source: Cell Death Dis. 2024 Aug 26;15(8):623. doi: 10.1038/s41419-024-07002-z (PMC11347571; doi:10.1038/s41419-024-07002-z)
Supplement: Supplementary file 1 — Supplemental Material 1 [file 41419_2024_7002_MOESM1_ESM.docx]

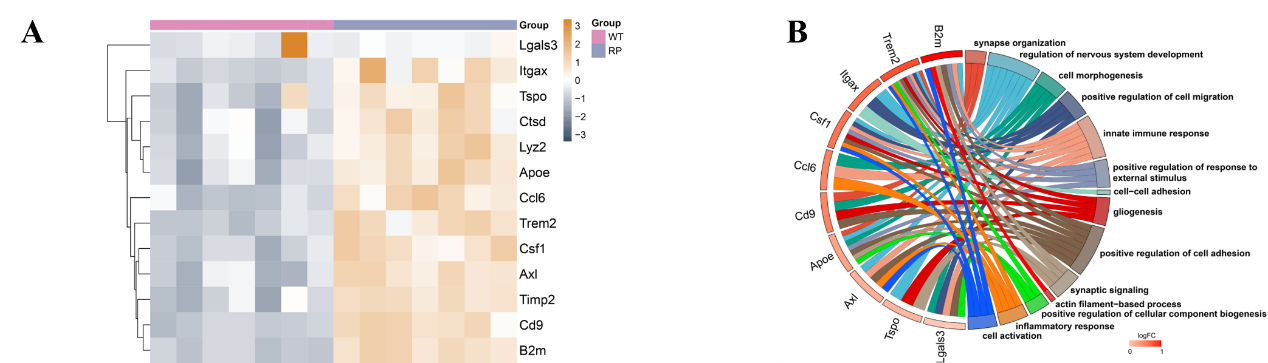


**Figure S1.** Retinal gene expression profile of *Rho^P23H/WT^* mouse (GSE152474 from GEO database). **(A)** Heatmap of DAM gene expression in *Rho^P23H/WT^* mouse. **(B)** The chord diagram of selected DEGs showing Trem2 as a major modulator involved in most affected pathways.


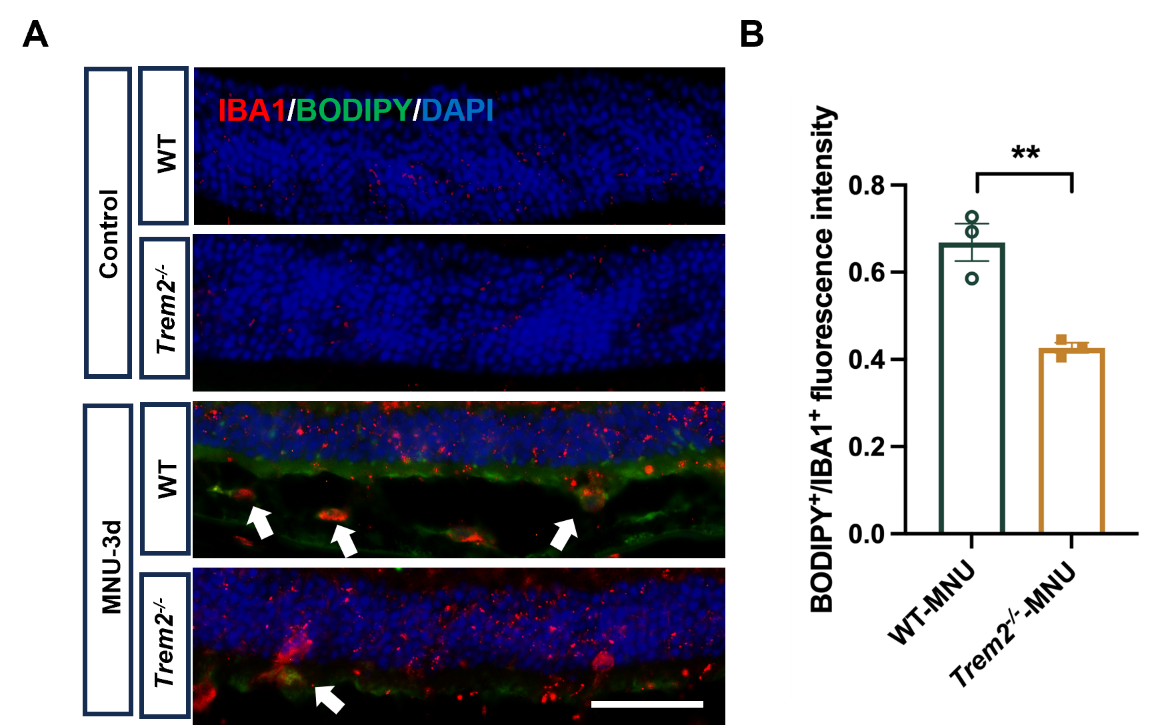


**Figure S2.** Lipid accumulation was decreased in Trem2-deficient microglia following MNU treatment. **(A)** BODIPY staining of retinal sections from MNU-induced WT and *Trem2^-/-^* mouse. **(B)** Quantitative analysis of BODIPY^+^/IBA1^+^ fluorescence intensity between WT and *Trem2^-/-^* mouse.


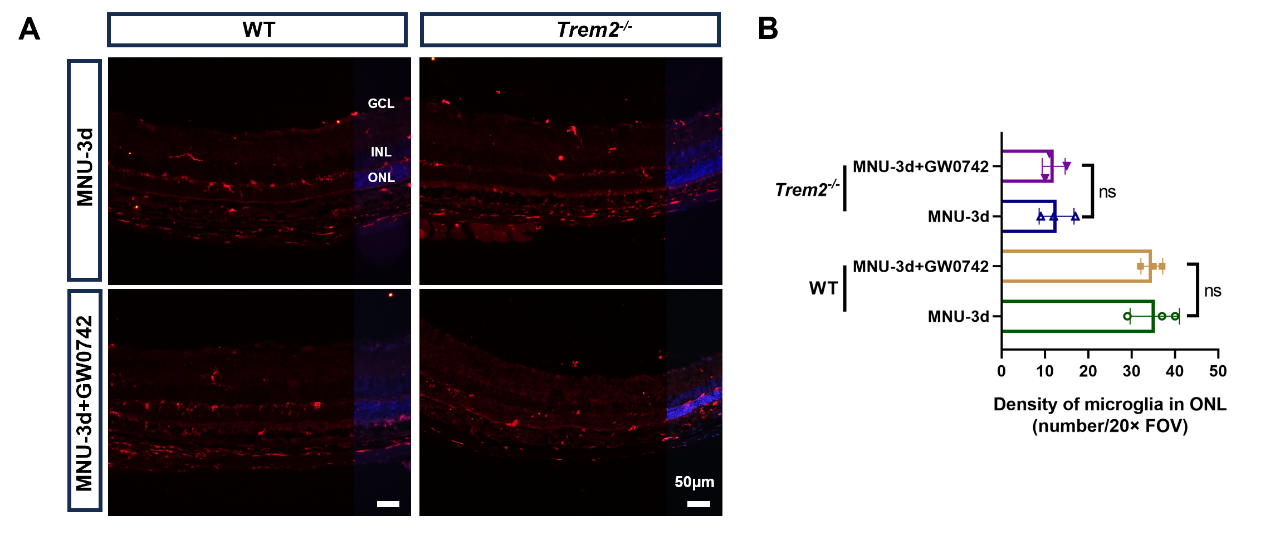


**Figure S3.** PPARβ/δ signaling was not significantly involved in MNU-induced photoreceptor degeneration. **(A)** Representative immunofluorescent images of IBA1-stained cells following GW0742 treatment in *Trem2^-/-^* and WT mice at 3 days post-MNU treatment. Scale bar, 50 μm. **(B)** Comparison of the infiltration and distribution of IBA1^+^ cells between WT and *Trem2^-/-^* mouse retina at 3 days post-MNU treatment and PPARβ/δ agonist treatment.


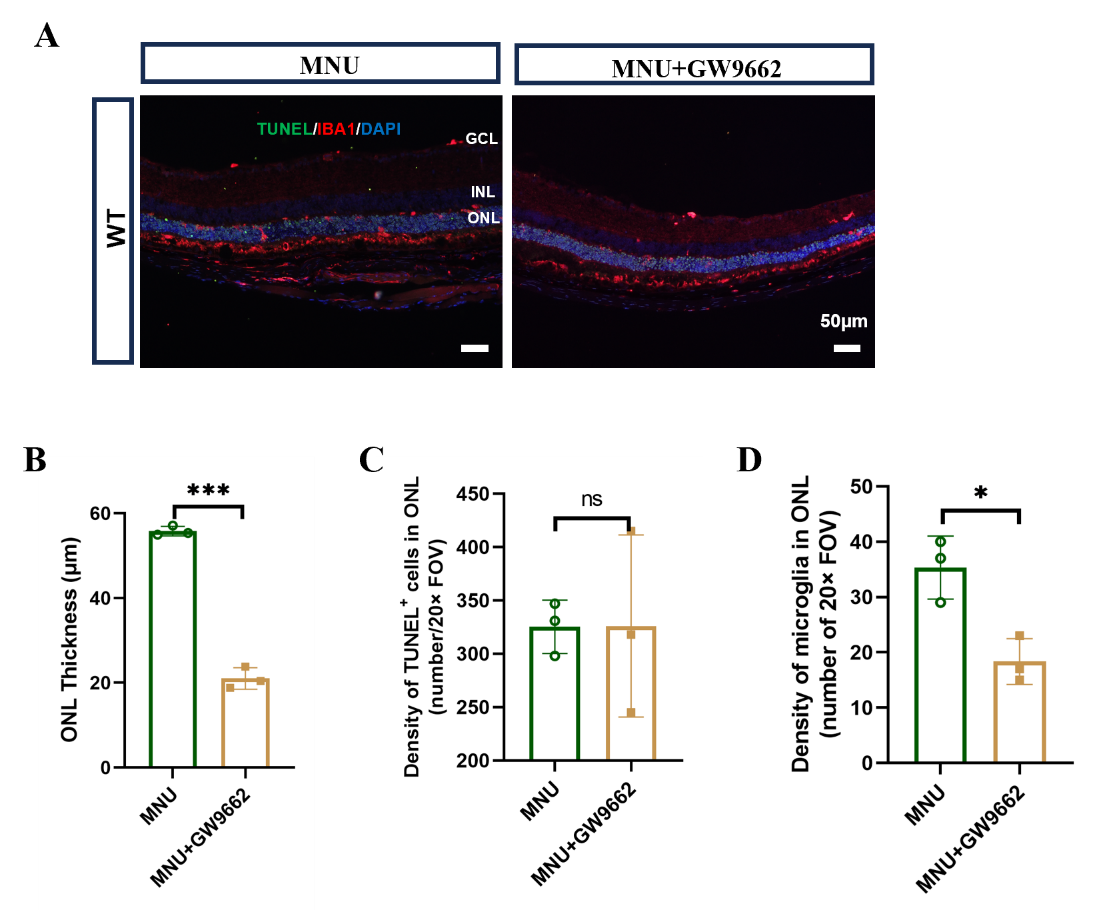


**Figure S4.** PPARγ inhibitor GW9662 exacerbated photoreceptor cell apoptosis and ONL thinning. **(A)** Representative immunofluorescent images of TUNEL- and IBA1-stained retina at 3 days post-MNU and GW9662 (PPARγ inhibitor) treatment in WT mouse. Scale bar, 50 μm. **(B, C)** Comparison of the ONL thickness **(B)** and number of TUNEL^+^ nuclei **(C)** between control and GW9662-treated WT mouse retina. **(D)** Comparison of microglial infiltration into ONL between control and GW9662-treated WT mouse retina.


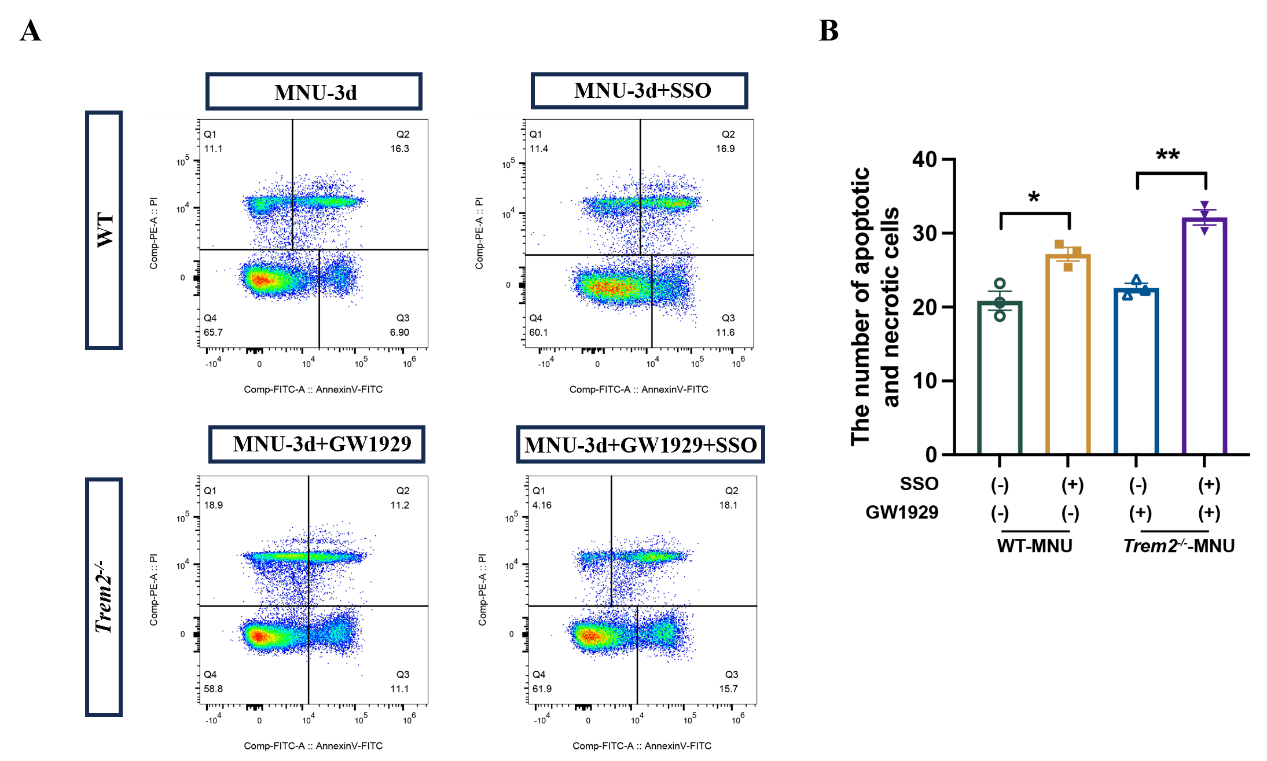


**Figure S5.** The co-administered GW1929 and SSO to MNU-treated *Trem2^-/-^* mice decreased accelerated photoreceptor degeneration compared to GW1929 treatment alone. **(A)** Flow cytometry analysis using Annexin V/PI staining. **(B)** Quantitative analysis of the number of apoptotic cells in GW1929-treated *Trem2^-/-^* mice with and without SSO.
