# Supplementary figures and images for: TREM2-dependent activation of microglial cell protects photoreceptor cell during retinal degeneration via PPARγ and CD36
Source: Cell Death Dis. 2024 Aug 26;15(8):623. doi: 10.1038/s41419-024-07002-z (PMC11347571; doi:10.1038/s41419-024-07002-z)

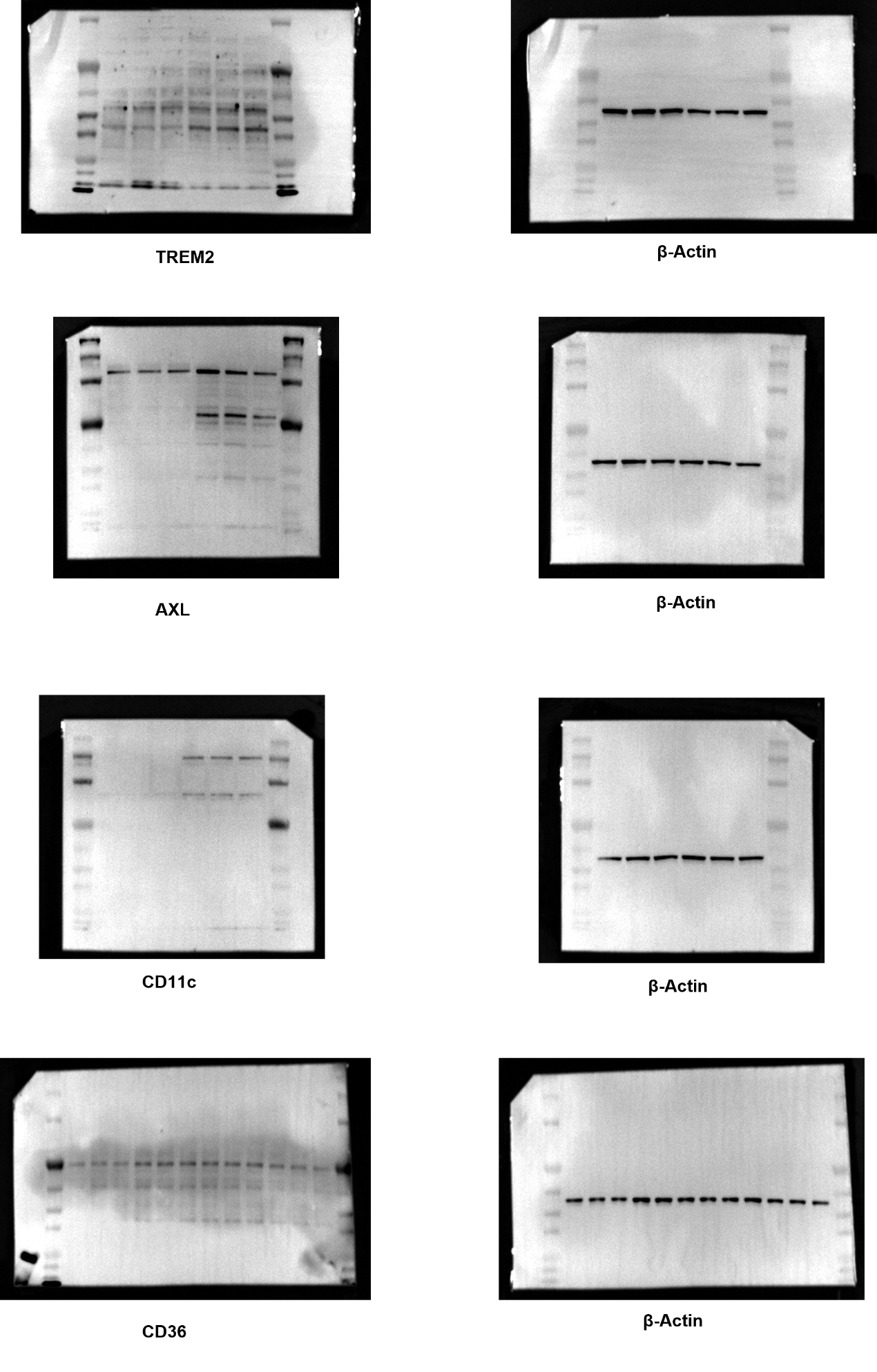


**Figure S1.** Full length uncropped original western blots used in this manuscript.

Supplement: Supplementary file 2 — Supplemental Material 2 [file 41419_2024_7002_MOESM2_ESM.docx]
